# Food self-provisioning: Implications for sustainable agroecological transition in rural Nigeria

**DOI:** 10.1016/j.heliyon.2024.e32098

**Published:** 2024-06-11

**Authors:** Chinasa Onyenekwe, Chukwuma Ume, Ebele Amaechina, Nice Chukwuma Ume, Ogochukwu Onah, Angela Obetta, Ejiofor Omeje

**Affiliations:** a Department of Agricultural Economics, University of Nigeria, Nigeria; b Institute of Agricultural Policy, Justus Liebig University, Giessen, Germany

**Keywords:** Agroecological transitions, Food self-provisioning, Sustainable agricultural practices, Farmer-driven solutions, Soil health and ecology

## Abstract

Agroecology is a sustainable farming method that has the potential to revolutionize the global agricultural sector by promoting cleaner and more environmentally friendly practices. However, the question of how to effectively transition to a sustainable agroecology system remains a topic of debate, particularly in developing economies. In many developing countries, subsistence farming plays a crucial role in supporting the livelihoods of countless households. Therefore, it is essential to explore the connection between food self-provisioning and the shift towards agroecology. Using primary data from rural Nigeria and by applying an ordered logistic regression, the study demonstrates that when farmers are primarily dependent on their own produce for sustenance, there is a natural inclination towards methods ensuring long-term soil health and ecological balance. We observed that self-provisioning leads to a 10.9 % increase in agroecology transition, and this result was statistically significant (P-value 0.001). This paradigm not only promotes sustainable agricultural practices but also underscores a holistic approach where agriculture coexists harmoniously with nature. As the global challenges of climate change and increasing food demand loom large, understanding and supporting these farmer-driven solutions become paramount. The results beckon policymakers and stakeholders to frame strategies grounded in farmers' intrinsic motivations, ensuring a sustainable agricultural future that is ecologically viable, culturally resonant, and economically beneficial.

## 1 Introduction

Food self-provisioning has long stood as a symbol of resilience and autonomy for numerous households across the globe. This practice, which is deeply entrenched in the tradition of producing food predominantly for household consumption, not only reinforces familial ties to agriculture but also underscores the primal connection between humans and the land they cultivate. Historically, this mode of sustenance has been a cornerstone in ensuring food security, especially in regions where the very act of procuring food from markets becomes a challenge [1] — be it due to physical or economic food access problems, or both (Ume et al., 2023). The essence of self-provisioning isn't just about growing food; it's about maintaining a degree of independence from volatile market forces and ensuring that the basic need for nutrition is met without being subject to external vagaries (Asfaw, Bantider, Simane et al., 2021).

Rural agrarian societies are emblematic of places where the rhythms of life are closely [2] entwined with the cycles of self-provisioning (Okidi, Ongeng, Muliro, 2022). In such localities, where the practice isn't just prevalent but is perhaps integral to the community's identity, delving into the intricate dynamics of self-provisioning becomes not just an academic endeavor but a societal imperative (Ume et al., [3]). The manner in which seeds are chosen, the patterns of planting, the techniques of cultivation, and even the rituals of harvest can provide profound insights into the socio-cultural fabric of the community and its symbiotic relationship with the environment [4].

Parallel to self-provisioning runs the expansive concept of agroecology (Ume et al., [5]). Rather than being a mere alternative to conventional farming, agroecology stands as a holistic approach to agriculture, one that seamlessly marries ecological principles with agricultural production. At its core, agroecology seeks to create a farming system that mimics natural ecosystems, leveraging their resilience and diversity [6]. Through practices like intercropping, where different crops are grown in proximity to benefit from each other, and agroforestry, which combines shrubs, trees, and crops in a single plot, agroecology champions diversity (Altieri & Nicholls, 2003). Then there's conservation agriculture, a method that emphasizes the protection of soil from erosion and degradation, and integrated pest management, which focuses on using beneficial organisms to control pests, minimizing the need for harmful chemicals [7]. Each of these practices, under the umbrella of agroecology, is aimed at achieving a singular vision: farms that are not only productive but are also sustainable, benefiting both the farmer and the environment. Soil health is rejuvenated, water is used more efficiently, greenhouse gas emissions are curtailed, and biodiversity thrives [8].

In essence, when one juxtaposes the idea of food self-provisioning with agroecology, the possibilities for synergy are boundless. The question then becomes: will emphasis on food self-provisioning help in the intensification of agroecology? How can these two activities inform, enrich, and perhaps even transform each other for a more sustainable and secure future? The intersection of self-provisioning and agroecology intensification presents a promising paradigm. On one hand, self-provisioning households, driven by the need for food security, may seek to adopt practices that ensure long-term sustainability and yield. On the other hand, the transition might be constrained by various factors, including access to knowledge, resources, or even cultural predispositions toward traditional farming methods.

Examining how food self-provisioning and agroecology interact can uncover potential synergies that drive sustainable farming practices and ensure food security. In the face of global challenges like climate change, soil degradation, and water scarcity, understanding how traditional and ecological farming methods can complement each other becomes a paramount concern [9]. For agrarian communities, where food self-provisioning is integral, delving into its intersection with agroecology provides invaluable insights into local practices, traditions, and knowledge. This exploration could pave the way for community-driven solutions that respect and uphold local traditions while seamlessly integrating modern sustainable practices. Additionally, if these activities can indeed mutually benefit each other, it could lead to a more efficient use of resources, ensuring maximum output with minimal input. Addressing this relationship not only offers a holistic perspective on agricultural practices but also places emphasis on environmental well-being, the socio-cultural dynamics of the community, and the long-term viability of these farming methods. Ultimately, the insights garnered from this study could significantly influence agricultural policies, guiding policymakers towards promoting practices that harmoniously blend ecological soundness with the rich traditions of self-provisioning. This confluence is pivotal in aiming for a future where agriculture remains sustainable and continues to guarantee food security. Thereby achieving one of the SDG goals.

The goal of this research is to investigate the determinants and implications of the transition to agroecological farming practices in rural communities. Specifically, it aims to understand the factors driving this transition, such as gender dynamics, land access, and self-provisioning strategies. The research seeks to shed light on how agroecology can contribute to sustainable agricultural development and food security. By examining the motivations and challenges faced by farmers, the study aims to provide valuable insights for policymakers, researchers, and practitioners working in the field of agriculture and rural development.

## 2 Food self-provisioning and agroecology transition

### 2.1 Food self-provisioning: an overview

Food self-provisioning, often referred to as subsistence farming or home gardening, has a rich history, especially in developing nations. In these regions, for generations, families have relied on their plots of land to sow and harvest crops, not primarily for trade or sale, but for their sustenance. Ume et al., 2022 and Vávra, Megyesi et al., [10] underscores this by pointing out how integral this form of agriculture has been in assuring that families have enough to eat. Especially in places where the infrastructure is still catching up or where the economy does not favor the common person, the act of going to a market to buy food is not just a simple chore. High prices, coupled with the difficulty of even reaching these markets due to transportation or logistical challenges, have made self-provisioning a necessity rather than a choice for many.

This perspective is not solitary. Ančić et al. [11] further echoed this view through their observations on the current dynamics of food consumption. Their studies revealed that by growing what they consume, households could circumvent the unpredictability and potential unreliability of market forces. In essence, having a garden or a small farm acts as a safety net. When markets fluctuate, when prices soar, or when supply chains are disrupted, these families have their provisions to fall back on. It is more than just about survival; it is about resilience and ensuring a level of self-sufficiency in an increasingly interconnected but unpredictable global economy.

### 2.2 Agroecology: A sustainable farming approach

Rooted in both scientific and indigenous knowledge, agroecology advocates for farming practices that work harmoniously with nature, rather than against it (Nicholls, 2016). By fostering greater biodiversity, enhancing soil health, and reducing dependence on chemical inputs, agroecology promotes a resilient and regenerative agricultural system. Moreover, it emphasizes community engagement and traditional knowledge, valuing the experiences of farmers and local communities in crafting solutions tailored to local contexts. The shift towards agroecological practices is not just about sustainable food production; it also represents a larger movement toward holistic land management, where ecological health, human well-being, and socio-economic equity converge (Nicholls, 2016). The overarching goal of agroecology is not just about harvesting crops, but about doing so in a manner that leaves the environment better off, or at the very least, unharmed. This philosophy recognizes the finite nature of our planet's resources and the need for sustainable agricultural practices that can support the ever-growing global population.

The beauty of agroecological practices lies in their diversity and adaptability (Ume et al., 2023). Methods such as intercropping, where different crops are planted in proximity, not only optimize the use of land but also reduce the need for chemical pesticides as certain plant combinations naturally deter pests [12,13]. Agroforestry, another innovative method, involves the integration of trees into farming systems [14]. This not only provides shade and protects the soil but also offers additional benefits such as fruit or timber. Conservation agriculture, on the other hand, focuses on soil health, ensuring it remains fertile and capable of supporting crops for generations to come (Mekuriaw, 2023). All these practices are bound by a common thread - they are nature-inspired, aiming to work in tandem with the environment rather than against it. Ume (2022) provides a more in-depth examination of these agroecological practices, echoing the sentiment that they could very well be the future of farming. The authors emphasized the resilience these methods offer, especially in the face of the ever-looming threat of climate change.

To sum up, the transition to agroecological practices isn't just a shift in farming techniques; it's a paradigm shift in how we perceive agriculture's relationship with the environment. It moves away from an extractive approach to one of coexistence, understanding, and respect for nature. As Altieri & Nicholls [15] suggest, if embraced widely, agroecology could steer the world towards a future where food production and environmental conservation are two sides of the same coin.

### 2.3 The intersection of food self-provisioning and agroecology

The convergence of self-provisioning and agroecology creates a unique paradigm, offering potential synergies. Very few studies have attempted to investigate this connection. Emeana et al. (2020) explored this intersection theoretically, postulating that while self-provisioning households might have a heightened incentive to adopt sustainable practices to ensure long-term food security, their transition might be constrained by factors such as access to knowledge, resources, and cultural predispositions toward traditional farming. Furthermore, Ume et al. (2023) highlighted the role of gender and socio-economic characteristics in determining the adoption of agroecological practices within self-provisioning settings. In essence, self-provisioning serves as an adaptive response to economic and logistical constraints, providing a safety net against food insecurity. Pungas (2019) resonates with this perspective, suggesting that self-provisioning has the added advantage of reducing the volatility associated with market fluctuations, thereby solidifying its role as a sustainable food procurement strategy.

Drawing these two strands of literature together reveals an intricate system of interconnections. The convergence of food self-provisioning and agroecology presents an innovative paradigm for the future of agriculture. Yotova [16] observes that when self-provisioning households adopt agroecological practices, they not only bolster their immediate food security but also contribute to longer-term environmental sustainability. In agrarian communities, this intersection could prove transformative, paving the way for an agricultural model that is both ecologically sustainable and economically resilient. Understanding and harnessing the synergies between food self-provisioning and agroecology can chart a path toward a more secure and sustainable future for global agriculture. However, there is no empirical evidence in literature showing that food self-provisioning is associated with agroecology intensification.

Although the academic and agricultural landscapes have witnessed increasing discussions on food self-provisioning and agroecology intensification. Yet, strikingly, there is a dearth of empirical research that directly connects these two concepts. Such an omission in the literature suggests uncertainty regarding the interplay between food self-provisioning and agroecological practices. While both emphasize sustainable food production systems, the nature of their interaction, be it complementary or contradictory, is not well understood. This gap in knowledge underscores the imperative to delve deeper into this intersection, evaluating the prospective advantages and potential hurdles of amalgamating these two approaches.

## 3 Methodology

### 3.1 Description of the study area

**Fig. 1:**
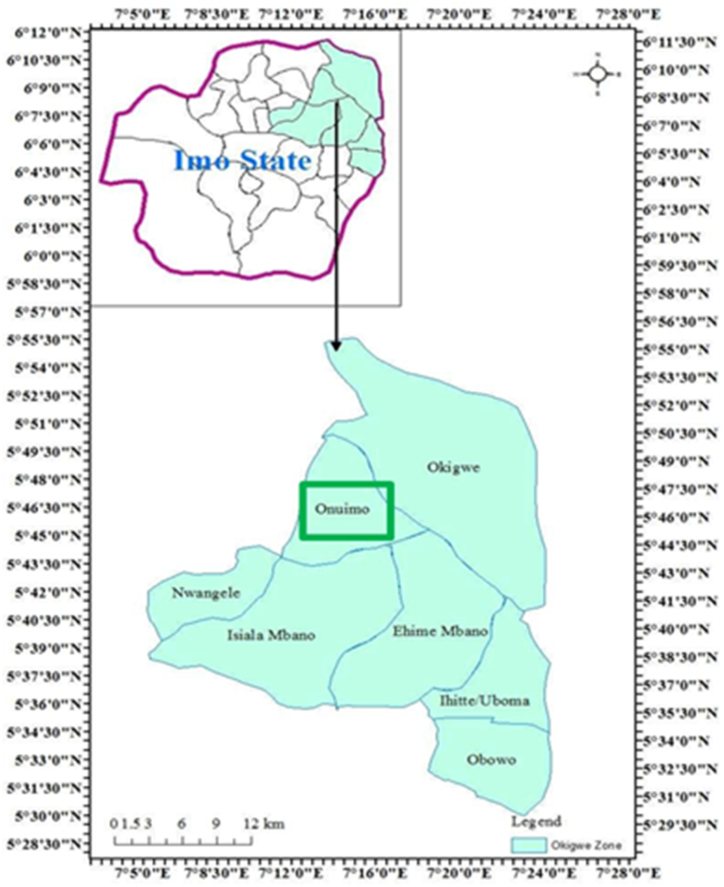
Map of Imo state showing the research area: Source [18].

Onuimo Local Government Area (LGA) in Imo state serves as the focal point of this study, as depicted in Fig. 1. Positioned in the North-Eastern part of Imo state, Onuimo covers a geographical expanse of approximately 87 km^2^ (54.0593). It stands as one of the twenty-seven distinct LGAs within Imo state, which is situated in the South-eastern geopolitical quadrant of Nigeria. Geographically, Onuimo can be pinpointed at coordinates 5° 46′ 25″ North and 7° 14′ 28″ East [17].

The reason behind choosing Onuimo LGA was based on the fact that it was where the researchers from the Center for Agroecology had established an agroecology group in 2016. The researchers had already established relationships and collaborations with farmers in this region through their work on promoting agroecological practices. This pre-existing connection allowed for easier access to primary data collection and ensured a higher level of trust and cooperation from the participants. Additionally, Onuimo LGA was chosen due to its representative nature as a rural farming community in Imo State. It encompasses various towns and villages, representing diverse farming practices and socio-economic backgrounds within Imo State. By selecting a specific area where agroecological initiatives were already underway, we aimed to better understand the dynamics of food self-provisioning and its implications for sustainable agroecological transitions in this context. This provides valuable insights into localized challenges, opportunities, and strategies that can inform future interventions not only within Onuimo LGA but also potentially across other similar rural communities.

Onuimo Local Government Area (LGA) of Imo state, with its administrative capital located in the town of Okwe, encompasses an expansive geographical spread that includes notable towns and villages such as Okwelle, Alaike, and Umuodumodu, among others. As per demographic data from [19], the LGA has an estimated population of approximately 99,368 inhabitants. The predominant ethnicity represented in this region is the Igbo group, and consequently, the Igbo language is the prevalent linguistic medium. Furthermore, in terms of religious affiliations, Christianity emerges as the dominant faith, a fact that underscores its sociocultural influence in the area.

In the area of agriculture, Onuimo LGA stands out for its agricultural productivity. The cultivation of crops such as oil palm, maize, yam, cassava, and a plethora of fruits and vegetables showcases the region's agricultural prowess. Beyond agricultural pursuits, the inhabitants of the LGA also engage in varied professions, including hunting, animal husbandry, and specialized trades like welding. This spectrum of occupations illustrates the multifaceted economic landscape of the LGA and its inhabitants' multifarious skill sets.

### 3.2 Case study description

Agroecology gained traction in Onuimo in 2016 through training programs facilitated by researchers from the center of Agroecology, United Kingdom together with the Information Resource Centers (IRCs) under the Research Extension Farmer Input Linkage Systems (REFILS). These initiatives aimed to promote the adoption of sustainable farming practices and the establishment of agroecology-oriented markets, with a particular focus on empowering smallholder farmers. Participation in these training programs was voluntary, leading to the formation of informal agroecology groups among farmers. These groups fostered knowledge sharing and collaborative action through peer-to-peer networks facilitated by a registered smartphone application, bridging scientific and traditional farming knowledge to enhance sustainable food production.

However, the emergence of a FADAMA project in the region, which prioritized rice production, led to the exclusion of food crop farmers and heightened governmental support for rice cultivation. Consequently, smallholder farmers were compelled to engage in rice production to access markets and essential resources. This shift marginalized farmers who sought to maintain autonomy over their food production. In response to this marginalization, agroecology groups emerged as a means of empowering farmers through collective action, resource pooling, and the development of local markets.

Furthermore, the study conceptualized agroecology farmers as those who identified with agroecology groups and adopted specific sustainable farming practices, including crop diversification, bio-fertilization, and natural pest control. In contrast, food insecurity was defined as a situation arising from inadequate physical or economic access to food. The study also explored the economic behavior of agroecology households, emphasizing the importance of household reproduction activities in ensuring food security through either on-farm production or engagement in market activities.

### 3.3 Study approach/design

The research design employed in this study is a cross-sectional design. This means that data was collected at a single point in time from different individuals or entities (farmers) within the study population. The use of a cross-sectional design allows for the examination of relationships between variables and provides a snapshot of the current state of affairs. Primary data was collected for this study, which means that researchers directly gathered information from participants through structured questionnaires. This approach enables researchers to collect specific and relevant data directly from the source, ensuring accuracy and reliability. Primary data collection also allows for flexibility in tailoring questions to address the research objectives.

The study utilized a quantitative approach, focusing on numerical data analysis and statistical methods to examine relationships between variables. This approach enabled researchers to quantify and measure key factors such as self-provisioning levels, agroecology adoption rates, gender dynamics, farm size, off-farm income, extension services utilization, and more. Through statistical analysis techniques like ordered logistic regression models and interaction effects analysis, quantitative data provides objective insights into patterns and trends within the dataset. Justification for using these approaches can be attributed to several reasons.

• Using primary data collection methods ensures that information is collected directly from participants without any intermediaries or potential biases.
• By designing structured questionnaires tailored to address research objectives, researchers can gather precise information necessary for examining relationships between food self-provisioning and agroecological transitions.
• The quantitative approach allows for objective measurement of variables using statistical methods which reduces subjectivity in interpretation.
• Utilizing primary cross-sectional data enables drawing conclusions about broader populations beyond just the sample studied due to its representative nature when appropriate sampling techniques are applied.

### 3.4 Sample size determination and sampling procedures

In order to ensure a representative sample of farmers practicing both conventional methods and agroecology, we employed cluster sampling. The clusters were defined based on the type of farming practice being followed – conventional or agroecology. To obtain a list of conventional farmers, we reached out to the Agricultural Development Program (ADP) regional headquarters, which provided us with a comprehensive list of farmers practicing conventional methods in the study area. Similarly, for the agroecology group, we collaborated with an agroecology facilitator who provided us with a list of farmers actively engaged in agroecological practices.

From these lists, we randomly selected 111 farmers from the agroecology group and 223 farmers from the conventional group, ensuring that each farmer had an equal chance of being included in the study. This resulted in a total sample size of 334 respondents. By utilizing cluster sampling and including both types of farming practices, our sample aimed to capture the heterogeneity within each group while maintaining homogeneity between them. This approach allowed us to compare and analyze data from both groups separately and assess any differences or similarities in their adoption of sustainable agricultural practices. This sampling method ensured that our study represented a diverse range of farmers practicing different farming methods while providing insights into their adoption levels and factors influencing their transition towards sustainable agriculture.

### 3.5 Data collection

The data collection process for this study involved administering a structured questionnaire to the participants. Trained enumerators, who were fluent in the local language, conducted face-to-face interviews with the respondents from 2021 to 2022. The data for this study was collected through a house-by-house survey conducted directly on the farms of the participants. Trained enumerators visited each household in rural areas of Nigeria's Onuimo Local Government Area (LGA) in Imo state to administer the questionnaire. The enumerators conducted face-to-face interviews with the farmers, visiting their plots of land to gather information on their agricultural practices and agroecology adoption.

The questionnaire was designed to gather information on various aspects related to the study. The first part of the questionnaire focused on demographic details, such as age, gender, education level, and occupation. It also included questions about asset ownership and access to services like extension visits. The second part of the questionnaire specifically targeted agroecology adoption levels. It included questions about specific crop practices, such as tillage methods, fertilization techniques, irrigation methods, and weed/pest/disease management approaches. The questionnaire distinguished between field/farm-level practices and landscape-scale practices. The purpose of collecting this data was to understand the determinants of agroecological transitions and explore how food self-provisioning is related to these transitions.

During the survey administration process, all respondents were informed that they had the option to decline answering any questions they were uncomfortable with. However, no objections were raised, and all 334 surveys were deemed useable for analysis."

### 3.6 Ethical consideration

Ethical considerations are an essential aspect of any research study involving human participants. In this study, specific measures were taken to ensure the ethical treatment of participants and protect their rights and confidentiality.

Firstly, a participant information sheet was provided to each participant before their involvement in the study. This information sheet outlined the purpose of the study, what participation would entail, any potential risks or benefits, and how their data would be used. Providing this information allowed participants to make an informed decision about whether they wanted to participate in the study. Explicit consent forms were also obtained from each participant. These consent forms clearly stated that participation was voluntary and that participants had the right to withdraw at any time without facing any consequences. By obtaining explicit consent, it ensured that individuals willingly agreed to participate in the study.

To maintain participant confidentiality and protect their privacy, identifying information such as names or addresses were not collected or recorded on questionnaires. Instead, each participant was assigned a unique questionnaire number for data analysis purposes. Additionally, village names rather than specific locations were used to provide context. Furthermore, ethical approval for conducting this research was obtained from the Ethics Committee of Imo State Postgraduate Committee with reference IMO/AGRO/202/A062. This approval ensures that appropriate ethical standards were followed throughout the research process. By adhering to these ethical considerations, this study ensured respect for participants' autonomy and protected their privacy while collecting valuable data for analysis purposes.

### 3.7 Data analysis

The data for the study were analysed using both descriptive and inferential statistics. As presented in Equation (1), level of self-provision was computed as follows:

(1) $$\text{Food}\;\text{self}-\text{provisioning}=\frac{Y}{Y-S}\;*\;100$$

Where Y is the total crop produced per hectare of farmland.

S is the total amount of crop sold.

Y – S is the amount produced per hectare that was consumed by the household.

In other to examine the determinants of agroecology adoption, ordered logistic regression model was employed. This is a regression model that generalizes logistic regression by allowing more than two discrete outcomes that are ordered. Ordered logistic model is used to model relationships between a polytomous response variable which has an ordered structure and a set of regressor variables. The standard ordered logistic model is widely used to analyze discrete data of this variety (e.g. Oladokun et al., 2018; Sujakhu et al., 2018; Onyenekwe et al., 2022) and is built around a latent regression expressed in Equation (2):

(2) $$y_{i}*=\beta_{0}+\sum_{\left(j=1\right)k}\beta_{\left(j\right)}\;x_{\text{ij}}+\mu_{i}$$

Where x and β are standard variable and parameter matrices, and ε is a vector matrix of normally distributed error terms. Obviously predicted grades (yi*) are unobserved. We do, however, three level threshold cut off points as represented in Equations (3), (4), (5):

(3) $$y_{i}=1\;\text{if}\;y_{i}*\leq 1$$

(4) $$y_{i}=2\;\text{if}\;1<\;y_{i}*\leq \mu_{1}$$

(5) $$y_{i}=3\;\text{if}\;\mu_{1}\;<\;y_{i}*\leq \mu_{2}$$

Where μ_1 and μ_2 are the cut points i.e. the threshold variables in the logistic model. The threshold variables are unknown and they indicate the discrete category that the latent variable falls into. They are determined in the maximum likelihood estimation procedure for the ordered logistic regression.

The likelihood for adoption by an individual is presented in Equations (6), (7)).

(6) $${\left[\varphi \;\left(0‐\;X_{i}\;\beta \right)\right]}^{\text{Zi}1}\;{\left[\varphi \left(\mu_{1}‐\;X_{i}\;\beta \right)‐\varphi (0‐X_{i}\;\beta \;\right]}^{\text{Zi}2}\;{\left[1‐\varphi \left(X_{i}\;\beta ‐X_{i}\;\beta ‐\mu_{1}\;\right)\right]}^{\text{Zi}3}$$

(7) $$Z_{\left(\text{ij}\right)}=1\;\text{if}\;y_{i}=j;0\;\text{otherwise}\;\text{for}\;j=0.1\;\text{and}\;2$$

Where for the i_th_ individual, y_i_ is the observed outcome and X_i_ is a vector of explanatory variables. The unknown parameters β_j_ are typically estimated by maximum likelihood.

**Fig. 2:**
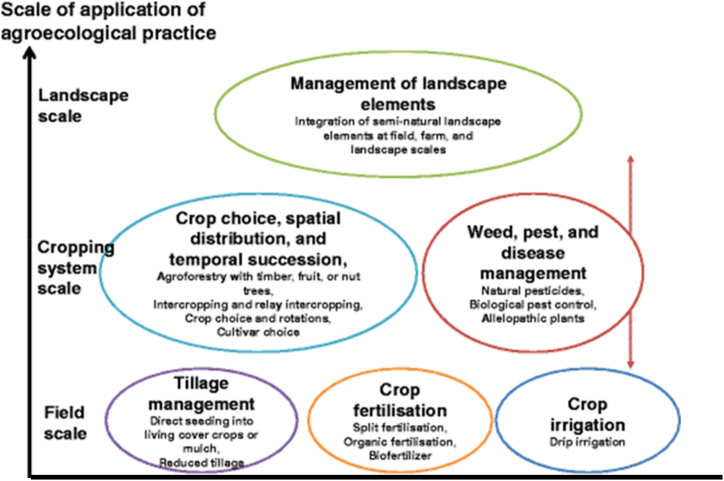
Different categories of agroecological practices. Source [20].

Y is the level of adoption of agroecological practices. Using the K-means cluster analysis, the agroecological practices employed by the farmer was categorized into low, medium and high which correspond to Field scale, cropping system scale and Landscape scale respectively (Fig. 2).

The application of the K-means cluster analysis offers a sophisticated method for segmenting and categorizing data based on inherent similarities. In the context of evaluating the agroecological practices adopted by farmers, this clustering technique was been employed to delineate these practices into distinct categories, specifically: low, medium, and high. These categories aren't arbitrary but are intrinsically tied to the scope and scale of farming operations (Ume & Bahta, 2024; Wezel et al., 2014).

The 'low' category corresponds to the Field scale, suggesting practices that are primarily localized and are concerned with individual plots or fields. Such practices might revolve around soil health, pest control, and crop variety within a singular field, and might be more reactive in nature, responding directly to the immediate needs of that specific plot. These include.

a) Tillage Management: It emphasizes reduced tillage practices, direct seeding into living cover crops, and the utilization of mulch to retain soil moisture and prevent erosion.
b) Crop Fertilization: Here, the focus is on sustainable and organic methods of nourishing the soil and crops. It highlights split fertilization, organic methods, and the use of biofertilizers that introduce beneficial microbes to the soil.
c) Crop Irrigation: Efficient water management is crucial, and this category highlights the importance of drip irrigation, which delivers water directly to plant roots, reducing water wastage.

The 'medium' category aligns with the Cropping system scale. This intermediate scale revolves around the choices and methodologies related to the crops themselves. Specific practices include.

a) Crop Choice, Spatial Distribution, and Temporal Succession: This focuses on the selection of crops, their spatial arrangement, and the order in which they are planted. Notably, it highlights the practice of agroforestry with trees, fruits, or nuts and the benefits of intercropping and relay intercropping.
b) Weed, Pest, and Disease Management: This underscores the importance of natural and ecological ways to manage pests and diseases. This includes the use of natural pesticides, biological pest control methods, and allelopathic plants that can naturally suppress weeds.

This implies a broader approach, where the practices aren't confined to a singular field but consider the interconnectedness of multiple fields or crop types. It encapsulates decisions related to crop rotation, polycultures, and intercropping—practices that acknowledge the synergistic relationship between different crops and their impact on soil and pest dynamics over a rotation period.

Lastly, the 'high' category is equated with the Landscape scale. This is the broadest scale focusing on the overall management of landscape elements. It emphasizes the integration of semi-natural landscape components at the field, farm, and broader landscape levels. This scale envisions the farm as part of a larger ecological system, ensuring its operations integrate harmoniously with the surrounding environment. The focus transcends individual plots or cropping systems. Instead, it emphasizes the larger agroecosystem, integrating practices that consider the well-being of the entire farming landscape. This could include considerations related to habitat conservation, watershed management, and integration of natural vegetation within farmlands. Such practices recognize the farm's place within a broader ecological context, emphasizing harmony with surrounding natural ecosystems and long-term sustainability.

In essence, by employing the K-means cluster analysis, a hierarchical understanding of agroecological practices is established, ranging from localized field-specific interventions to holistic, landscape-wide strategies, thereby offering farmers a structured approach to enhancing their agricultural productivity and sustainability. Table 1 presents the definitions and descriptive statistics of the variables used in the model.

**Table 1:** Definition and descriptive statistics of exogenous, outcomes and control variables.

Variables	Description
**Outcome variables**	
Agroecology adoption	Level of adoption of agroecological practices by household (dummy)
**Variables of interest**	
Gender	Male = 1, Feale = 0 (dummy)
Self-provisioning	% of food consumed by a farmer that is produced by the farmer (%)
***Socioeconomic characteristics***	
Occupation	Main occupation of the farmer (1 = Farming; 0 = Other occupations)-dummy
Family size	Number of individuals in a household eating from the same pot (number)
Farm size	Size of land under cultivation (Ha)
Farming experience	Number of years in farming (years)
Tropical Livestock Unit^a^	livestock from various species converted to a common unit (is it an index)
Off-farm income	Money gotten from non-farm undertakings, gifts or cash transfers ('000 Naira)
No. of relatives	Number of close family the farmer can depend on at difficult times in a community (number)
***Access to development services***	
Extension visits	Number of extension visits in the last farming season (number)
Credit constrained	If applied for credit but did not receive or receive less than it applied for, credit-constrained (1 = Yes; 0 = No) (dummy)
**Instrumental variables**	
Local market participation	Percentage of marketed produce sold at the local market (%)
Education difference	Level of education attained by adult female and male (male-female)
Distance to market	Time taken to reach preferred selling point
*Location fixed effects*	
Umuduru-Egbeaguru	
Umuna	
Okwe	
Okwelle	

a The Tropical Livestock Unit (TLU) is a standard unit used to quantify and compare different types of livestock based on their live weight and nutritional needs. It is typically computed by assigning a standard weight of 250 kg to one TLU, which allows for the aggregation and comparison of various livestock species, such as cattle, sheep, and goats, based on their equivalent weight in TLUs.

## 4 Results

The findings of the study are presented under the following headings: Demographic and Socioeconomic characteristics of the respondents, Level of self-provisioning between male and female-headed farm households, Level of agroecology adoption between male and female-headed farm households, Association between food self-provisioning and level of agroecology adoption.

### 4.1 Demographic and Socioeconomic characteristics of the respondents

Table 2 provides a detailed comparison between agroecology and non-agroecology (Conventional) farmers across various socioeconomic characteristics. In terms of gender distribution, there were 19 male and 92 female farmers in the agroecology group, compared to 166 male and 51 female farmers in the non-agroecology group. The *t*-test result indicates that there is no statistically significant difference in gender distribution between the two groups (p = 0.097). This finding aligns with previous research by Ume et al. (2023), which also showed similar gender distributions among agricultural practitioners in rural settings.

**Table 2:** Comparison of Socioeconomic/Demographic Characteristics between Agroecology and conventional Farmers.

**Cate**gory	Agroecology	Conventional	T-test/Chi-square
**Gender**			
**Male**	19	166	t = −1.68, p = 0.097
Female	92	51	
**Educational Status**			
**No formal education**	14	25	t = 0.51, p = 0.615
**Primary school**	32	67	
**Secondary school**	35	70	
**University**	20	45	
Postg**raduate**.	10	16	
**Method of Land Access**			
**Ownership**	9	100	Chi-square (16.82), p < 0.001
**Rented**	28	50	
**Communal**	30	55	
Borrowed	44	12	
**Organic Farming**			
**Organic**	27	3	Chi-square (28.62), p < 0.001
**In-organic**	3	59	
Combination	81	161	
**Farming as Major Occupation**			
**Yes**	80	111	Chi-square (1.34), p = 0.247
No	31	112	
**Marketing Channel**			
**Direct sales**	40	70	Chi-square (3.91), p = 0.048
**Local market**	20	50	
**Main market**	30	80	
**Sales to intermediaries**	21	23	

Regarding educational status, the data reveals the number of farmers at different educational levels, ranging from no formal education to postgraduate degrees. The *t*-test examines whether there are significant differences in educational attainment between agroecology and non-agroecology farmers for each category. Ume et al. (2023) demonstrated a positive correlation between higher education levels and adoption of sustainable agricultural practices, which complements our findings regarding educational attainment among agroecology farmers.

In the method of land access category, the table compares how farmers in both groups access land for farming, including ownership, rental, communal arrangements, or borrowing. The chi-square test results suggest a significant difference in the method of land access between agroecology and non-agroecology farmers (p < 0.001). This underscores the importance of land tenure systems in shaping agricultural practices, as highlighted in previous studies by Kerr et al. (2018).

Examining organic farming practices, the data reveals the number of farmers practicing organic, inorganic, or a combination of both methods in each group. The chi-square test shows a significant difference in organic farming practices between agroecology and non-agroecology farmers (p < 0.001). This finding is consistent with the literature, which suggests that agroecology farmers are more likely to adopt organic farming methods due to their focus on environmental sustainability ([21]; Ume & Bahta, 2024).

Finally, considering whether farming is the major occupation, the comparison indicates the number of farmers for whom farming is the primary occupation versus those for whom it is not. This section also includes a chi-square test to assess any significant differences between the two groups (Ume, 2023). This finding highlights the diverse livelihood strategies adopted by farmers in rural areas and emphasizes the need for policies that support agricultural diversification (Kerr et al., 2018). Overall, this table offers a comprehensive overview of how agroecology and non-agroecology farmers differ across various socioeconomic characteristics, providing valuable insights for understanding agricultural practices and informing policymaking. It underscores the importance of considering contextual factors and local realities in designing interventions aimed at promoting sustainable agriculture [22].

### 4.2 Level of self-provisioning based on the gender of the households

In analyzing gender dynamics related to food self-provisioning in rural households, the result in Table 3 provides enlightening insights. Using the metric that determines the proportion of produce retained for household consumption as a percentage of the total produced, it becomes evident that gender plays an influential role in agricultural consumption decisions. For instance, female-headed households (head of house here entails that it is the woman that is in charge of farming in the household) display a notable propensity to retain a higher percentage of their maize produce, with a 90 % self-provisioning rate, compared to 60 % in male-headed households. This findings supports prebious studies such as Nelson (2004) and Roseman (2002). This distinction may suggest that maize is more of a dietary staple for female-led households.

**Table 3:** Comparison of Crop Self-Provisioning Rates in Female and Male farmers.

Crop	Female	Male	T-test value	Sign**.** level
Maize	90	60	2.56	0.032
Rice	98	85	3.15	0.012
Cassava	68	23	5.28	0.001
Pumpkin	31	10	4.12	0.005
Waterleaf	55	23	3.09	0.011
Okra	91	40	3.98	0.002
Red Pepper	53	10	4.45	0.003
Yellow Pepper	82	64	2.72	0.021
Yams	95	23	5.61	0.001
Bitter Leaf	90	60	2.54	0.031

Similarly, rice sees a striking 98 % retention rate for consumption within female farmers, overshadowing the still commendable 85 % in male-led households.

Further nuances emerge when examining cassava and pumpkin. Female-headed households retain a robust 68 % of cassava and 31 % of pumpkin they produce, compared to male counterparts who retain a mere 23 % and 10 %, respectively. This pattern extends to leafy vegetables and peppers as well, where crops like waterleaf, okra, red pepper, and yellow pepper all have significantly higher retention rates in female-headed households. Specifically, waterleaf sees a disparity of 55 %–23 %, okra at 91 %–40 %, red pepper at 53 %–10 %, and yellow pepper at 82 %–64 %, with female-led households consistently prioritizing these crops for household consumption over male-led ones.

Lastly, the data regarding tubers and local greens like yams and bitter leaf further amplifies this trend. An overwhelming 95 % of yams produced in female-led households are retained for consumption, dwarfing the 23 % seen in male-led households. Similarly, bitter leaf has a 90 % retention rate in female-led households compared to 60 % in male-led ones. In conclusion, the result underscores that gender exerts a considerable influence on decisions surrounding which crops are grown primarily for household consumption. Through the lens of the food self-provisioning metric, the findings highlight the dominant role of female farmers in promoting household food security and diversifying diets in rural settings.

**Fig. 3:**
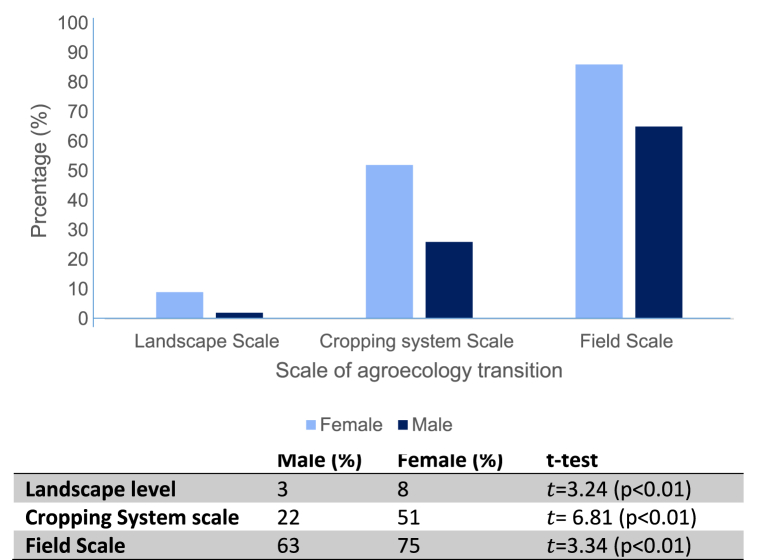
Percentage distribution of respondents based on level of Agroecology adoption.

The *t*-test results reveal significant disparities in self-provisioning rates between female-headed and male-headed households across various crops. For maize, rice, cassava, pumpkin, waterleaf, okra, red pepper, yellow pepper, yams, and bitter leaf, the self-provisioning rates were consistently higher in female-headed households compared to their male-headed counterparts. These differences were statistically significant for all crops, as indicated by the *t*-test values and corresponding significance levels. The findings suggest that gender plays a significant role in determining which crops are prioritized for household consumption, with female farmers demonstrating a greater propensity to retain produce for domestic use. This underscores the crucial contribution of female farmers to household food security and dietary diversity in rural settings. The observed disparities highlight the importance of gender-sensitive approaches in agricultural development interventions aimed at promoting food security and nutrition.Fig. 3: Percentage distribution of respondents based on level of self-provisioning.

### 4.3 Level of agroecology adoption between male and female-headed farm households

The results showing the level of agroecology adoption between male and female-headed households is presented in Fig. 3. The result illustrates the transition to agroecological practices among female and male farmers across three defined scales: Landscape Scale, Cropping System Scale, and Field Scale.

#### 4.3.1 Landscape Scale

At the Landscape Scale, a relatively small percentage of both male and female farmers have transitioned to agroecological practices. However, the adoption rate among female farmers is notably higher than their male counterparts. This suggests that at the broadest level, which concerns the holistic integration of semi-natural landscapes into farming practices, both genders exhibit a degree of hesitancy or face challenges. The reasons for this could be manifold, ranging from lack of awareness, access to resources, or the perceived high initial investment in transforming large swaths of land [23].

#### 4.3.2 Cropping system scale

The Cropping System Scale reveals a more pronounced difference between male and female adoption rates. While both genders show an increased percentage compared to the Landscape Scale, male farmers demonstrate a considerably lower adoption rate. This scale, which emphasizes crop choices, spatial distribution, temporal succession, and ecological pest management, appears to resonate more with male farmers. One might infer that female farmers may have better access to information regarding crop rotation, intercropping, or agroforestry [24]. Alternatively, they might be benefitting from networks or cooperatives that promote such practices.

#### 4.3.3 Field scale

The Field Scale represents the granular, ground-level practices, focusing on tillage, fertilization, and irrigation techniques. Interestingly, this is where both genders show the highest adoption rates, but with a twist—female farmers surpass males. Female farmers seem to excel in applying sustainable practices directly on the field. This could imply that female farmers, often responsible for direct cultivation tasks, are more inclined to embrace sustainable field-level practices (Ume et al., [25]). They might find techniques like reduced tillage, organic fertilization, or drip irrigation more feasible or beneficial in their daily operations. The near-equal percentages for males indicate that they too see the value in these practices, but it is intriguing to note the lead females take at this level.

#### 4.3.4 General observations

There is a clear progressive increase in the adoption of agroecological practices from the Landscape Scale to the Field Scale for both genders. This might indicate that farmers find it easier or more immediately beneficial to implement ground-level changes before transitioning to broader landscape adjustments.

The differential adoption rates between genders emphasize the need for gender-sensitive training and resource provision. Tailoring outreach and training programs to address the unique challenges and priorities of each gender can lead to more uniform adoption rates across all scales. The result underscores the dynamic interplay between gender and the adoption of agroecological practices. It highlights areas where targeted interventions can bridge the gap, ensuring that both male and female farmers are equally equipped to transition towards sustainable agriculture.

The *t*-test results reveal significant differences between male and female groups across various scales of analysis. At the landscape level, there was a statistically significant difference (*t* = 3.24, *p* < 0.05), with female-headed households reporting a higher mean compared to male-headed households. Similarly, at the cropping system scale, the mean for female-headed households was significantly higher (*t* = 6.81, *p* < 0.01), indicating a substantial discrepancy in agricultural practices between the two groups. Furthermore, at the field scale, the *t*-test also showed a significant difference (*t* = 3.34, *p* < 0.05), with female-headed households exhibiting a higher mean compared to their male counterparts. These findings underscore the gender-based disparities in agricultural activities, suggesting that female-headed households tend to engage more extensively across different scales of farming practices compared to male-headed households.

### 4.4 Determinants of agroecology adoption

The results of the ordered logistic regression analysis present a multifaceted exploration into the determinants shaping the agroecological transition. Several variables, based on their coefficients and p-values, stand out as significant influencers, while others don't exhibit a marked impact (Table 4). Interestingly, gender does not significantly influence the transition level, given its p-value of 0.721. This is contrary to some studies that emphasize gender disparities in agricultural practices [26]; [27]; [28]. However, this finding might suggest that when given equal opportunities and resources, both genders are equally likely to transition in their agroecological practices.

**Table 4:** Marginal effect of the Determinants of agroecology transition.

Variables	Coefficient	t-value	P-value
Number of agroecology adopted	0.7442	2.33	0.020**
Gender	−0.11004	−0.36	0.721
Self-provisioning	10.9379	6.24	0.000***
Occupation	−0.3631	−0.40	0.688
Family size	−1.5112	−0.87	0.386
Farm size	−1.7799	−2.04	0.041**
Farming experience	−0.23685	−1.39	0.166
Tropical Livestock Unit	−0.0001	−0.08	0.937
Off-farm income	4.1491	4.66	0.0017***
No. of relatives	0.0411	1.51	0.131
Extension visits	1.8960	3.96	0.0021***
Credit constrained	0.0004	0.20	0.845
Local market participation	−0.1529	−0.58	0.561
Education difference	−0.14205	−0.24	0.807
***Interaction effect***			
Self-provisioning * Gender	−0.2322	−2.14	0.051**
Self-provisioning * Farm size	−2.8541	−8.99	0.000***
Self-provisioning * Off-farm income	8.1141	3.48	0.050**
Self-provisioning * Credit	0.0105	0.92	0.2412
Self-provisioning * Extension visit	1.0278	6.12	0.000***
Self-provisioning * Education	−0.8751	1.01	0.2109
***Location fixed effect***			
Umuduru-Egbeaguru	0.0060319	0.01	0.992
Umuna	−0.7372187	−1.24	0.215
Okwe	−0.4522164	−0.76	0.450
Okwelle	0.0001756	0.06	0.956
Cut 1 −2.5485 2.6546			
Cut 2 −1.1152 2.6541			
Cut 3 −0.6352 2.6530			
No of observations 334			
LR chi2 (13) 121.48			
Prob > chi2 0.0011			
Pseudo R2 0.4122			
Log likelihood −429.652456			

With a notably high coefficient of 10.93719 and a significant p-value of 0.000, self-provisioning stands out as a strong predictor. This indicates that farmers who rely heavily on their produce for sustenance are more inclined to adopt sustainable practices. This is consistent with literature that identifies self-provisioning and food security as primary motivators for agroecological transitions [1,29,30]. The negative coefficient of −1.779945 with a p-value of 0.041 suggests that as the farm size increases, the likelihood of transitioning to higher levels of agroecology decreases. This might be due to the challenges of managing larger plots and the perceived risks associated with changing established practices. Larger farms might also be more commercialized and less inclined to change their methods. Farmers with additional sources of income outside their farm are more likely to transition to higher levels of agroecological practices. This is highlighted by a coefficient of 4.149124 and a highly significant p-value of 0.000. Additional income can provide the financial buffer needed to experiment with new practices without the immediate pressure of farm profitability. A positive coefficient of 1.896069 and a p-value of 0.000 underscore the pivotal role of agricultural extension services. Farmers who receive more extension visits are more likely to transition to higher levels of agroecology [31]. This emphasizes the importance of knowledge transfer and on-ground training, consistent with literature that supports extension services as a critical driver for sustainable agricultural transitions.

Other variables like occupation, family size, farming experience, and local market participation don't show a significant influence on the level of agroecology transition. Similarly, the regions of Umuodu-Ebegaanru, Umuana, Okwe, and Okwelle, based on their p-values, don't seem to exhibit a significant impact. The findings highlight the complex interplay of economic, social, and educational factors in influencing agroecological transitions. While some variables reinforce conventional wisdom and align with the broader literature, others offer fresh perspectives and challenge existing assumptions. As agroecological practices gain traction globally, such insights are invaluable in shaping interventions and policies that cater to the unique dynamics of specific farming communities.

The results of interaction effects shed light on the relationship between the levels of agroecology transitioning, and the interacting factor "Self-provisioning." The analysis considers various determinants, exploring how their interactions with self-provisioning influence the likelihood of transitioning to different agroecological levels. Notably, the interaction with gender exhibits marginal significance (p = 0.051), implying that the impact of self-provisioning on agroecology transitioning may vary between genders. A highly significant interaction is observed with farm size (p < 0.001), suggesting a substantial influence of self-provisioning on the likelihood of transitioning to different agroecological levels, especially for individuals with smaller farms. Additionally, the interaction with off-farm income is marginally significant (p = 0.050), indicating that the effect of self-provisioning on agroecology transitioning is more pronounced for those with higher off-farm income. On the other hand, interactions with credit constraint (p = 0.2412) and education difference (p = 0.2109) are not statistically significant, suggesting that self-provisioning's impact remains consistent irrespective of credit constraints or educational disparities.

In the broader context, these findings offer valuable insights for designing targeted interventions to promote agroecological transitions. The varying impact of self-provisioning based on gender, farm size, and off-farm income underscores the need for tailored strategies to accommodate diverse socio-economic contexts. Additionally, the non-significant interactions with credit constraint and education difference emphasize the robustness of self-provisioning as a determinant across different economic and educational backgrounds. This nuanced exploration of interaction effects enhances our understanding of the factors influencing agroecological transitions, providing valuable guidance for policymakers and practitioners involved in promoting sustainable agricultural practices.

## 5 Discussion and policy implications

The determinants underpinning the agroecological transition, as identified in the study, hold profound implications for the trajectory of sustainable agriculture. Drawing parallels with existing literature provides a comprehensive understanding of the dynamics at play. Starting with the transition rate of agroecological practices, it's evident that farmers who have integrated more of these sustainable practices are progressively aligning with comprehensive agroecological systems. Altieri (1987) and Gliessman (2014) previously highlighted this phenomenon, emphasizing that the amalgamation of multiple agroecological practices could lead to enhanced resilience and holistic productivity in agricultural systems. Such findings are hardly surprising given that the experience and success with certain agroecological methodologies can act as a stepping stone, encouraging farmers to further invest time and resources into broader sustainable practices.

In terms of gender dynamics, the study unveils an aspect that challenges conventional understanding. Historically, significant literature, notably by Agarwal (1992) and Doss (2002), underlined the gender disparities in agricultural spheres, from decision-making processes to resource allocation. However, the present research seems to align more with the recent discourses, such as the one presented by Peterman et al. (2014). These discourses suggest that as societies transition towards more egalitarian norms and resources become more accessible across genders, the role of gender as a defining factor in agricultural transitions could diminish. This shift in paradigm is not only indicative of broader societal progress but also highlights the evolving dynamics in the agricultural sector.

The relationship between self-provisioning and agroecological transitions emerges as another pivotal finding from this study. At its core, the principle is straightforward: when farmers rely predominantly on their produce for sustenance, there's an implicit drive to ensure the health of the soil and broader ecosystem. Rosset et al. (2011) resonate with this perspective, underlining how food sovereignty and self-sufficiency often become the propelling forces for farmers to gravitate towards agroecological methodologies. It's an intuitive understanding – a farmer deeply reliant on his land for sustenance would inherently seek practices that promise long-term yield and soil health.

The nexus between self-provisioning and agroecological transitions is undeniably crucial in the broader landscape of sustainable farming. Self-provisioning, which essentially means a farmer's reliance on his own produce for day-to-day needs, serves as a robust incentive for ensuring sustainable agricultural practices. The reason is twofold: first, there is a direct personal investment in the quality and health of the produce, and second, a recognition of the interconnectedness between the soil's health, the ecosystem, and the yield (Altieri, 1995). As one delves deeper into this association, it becomes clear that such farmers, who depend substantially on their farms for sustenance, inevitably look beyond the immediacy of high yields. Instead, their focus tends to shift towards ensuring the fertility and health of their lands in the long run (Gliessman, 2014).

The argument is not just empirical but also inherently logical. The commitment to self-sustenance engenders a protective and nurturing approach towards the land (Wezel et al., 2009). Here, the land is not merely an instrument for commercial profit but a lifeline, a provider of essential sustenance. Rosset et al. (2011) have elaborated on this very phenomenon, suggesting that when food sovereignty converges with the goal of self-sufficiency, farmers are often nudged towards adopting agroecological practices. This trend isn't just about securing food but also about preserving the very essence of farming – the soil and its health. Over time, this perspective helps to foster a holistic approach to farming, where short-term gains are weighed against long-term sustainability (Gliessman, 2014).

Furthermore, this nuanced understanding bridges the gap between immediate needs and future security. By relying on their own produce, farmers are not just feeding themselves but also laying the foundation for a sustainable agricultural future (Tilman et al., 2002). As global agricultural patterns experience dramatic shifts, and as climate change continues to pose unprecedented challenges, such agroecological transitions could be the cornerstone for ensuring both food security and environmental conservation (Godfray et al., 2010). The intertwining of self-provisioning with sustainable agricultural practices underscores a future of farming that is rooted in symbiosis — between the farmer, the land, and the ecosystem. It serves as a reminder that in the grand tapestry of agriculture, immediate gains and long-term sustainability are not mutually exclusive but can coexist harmoniously when guided by the principles of self-reliance and agroecology (Mendez et al., 2013).

One of the more intriguing revelations is the inverse relationship between the size of a farm and the inclination towards agroecological transitions. Drawing parallels from Mendez et al. (2017), it becomes evident that larger farms, often driven by commercial interests and short-term profitability, lean heavily towards industrial farming techniques. Such farms often prioritize immediate yield over long-term sustainability. In stark contrast, smaller farms, especially those managed by families, are more amenable to sustainable practices. The direct correlation between the health of the farm and the well-being of the family becomes the driving factor here.

The role of off-farm income emerges as a nuanced yet vital aspect in the broader narrative. As postulated by Barrett et al. (2001), having an alternate source of income can significantly diminish the financial risks associated with experimenting with new farming techniques. With the economic safety net that off-farm income provides, farmers are more likely to venture into innovative, sustainable practices, knowing that their immediate livelihood is not at stake.

Lastly, the transformative potential of extension visits cannot be overstated. Building on the insights by Feder et al. (2004), it is clear that bridging the chasm between academic agricultural research and real-world practices is crucial. Extension services, through personalized, on-site training, play an indispensable role in this, catalyzing the shift towards agroecological practices.

The intersections of individual motivations, socio-economic dynamics, and broader environmental considerations paint a holistic picture. As global agricultural paradigms grapple with the dichotomy of sustainability and immediate productivity, research endeavors like the present one provide a beacon, underscoring the importance of adaptive, context-centric approaches. The findings from this study offer vital policy implications, especially in the context of sustainable agriculture.

The findings of this study accentuate the link between food self-provisioning and the transition to agroecological practices. Recognizing this relationship has several policy implications, underscoring the need for a multifaceted approach to drive sustainable agricultural initiatives. Central to the recommendations is the promotion of localized farming systems. This aligns with the observation that when farmers' sustenance is directly tied to their land, they are more inclined towards sustainable practices that ensure long-term soil health and yield. Furthermore, by fostering educational and training programs, farmers can gain insights into the long-term benefits of agroecology, reinforcing their commitment to such methods. Financial incentives, like subsidies, can act as catalysts, making the shift to sustainable practices more economically viable for farmers. Investing in research ensures the development of tailored agroecological solutions, resonating with the study's emphasis on the significance of region-specific approaches. Strengthening food sovereignty echoes the study's findings on self-reliance, as safeguarding local agricultural systems ensures that communities have control over their food sources. In essence, the policy recommendations emanate from the study's core findings, emphasizing a holistic approach to encourage the intertwining paths of self-provisioning and agroecological transitions.

In interpreting the insignificance of certain variables in our model results (Table 3), several factors should be considered. Firstly, the absence of significance for certain variables may be attributed to the complexity of agricultural systems, where numerous interrelated factors influence outcomes. As highlighted by Adams et al. (2021), agricultural practices are often context-dependent and influenced by a multitude of socio-economic, environmental, and institutional factors. Additionally, the insignificance of certain variables may reflect the limitations of our dataset or the specificities of our study context. For example, the lack of significance for access to extension services may be due to variations in the quality and reach of extension programs, as noted by Asfaw et al. (2021). Furthermore, the non-significance of certain variables could also indicate potential multicollinearity issues or measurement errors, as cautioned by Mulugeta & Heshmati (2023) and Mgendi (2022). Therefore, while our model results provide valuable insights, further research incorporating broader datasets and accounting for contextual nuances is warranted to fully elucidate the determinants of agricultural outcomes.

In addition to the policy implications highlighted in the preceding discussion, it is essential to consider additional measures that can further incentivize the adoption of sustainable agricultural practices. Taxation policies, such as imposing taxes on environmentally harmful practices while offering tax credits for sustainable farming methods, can effectively steer farmers towards agroecology. By internalizing the environmental costs associated with conventional farming techniques, taxes create economic incentives for farmers to transition towards more sustainable alternatives. Moreover, the establishment of stringent environmental standards can serve as a regulatory framework to ensure adherence to sustainable practices and mitigate negative environmental externalities. Marketable permits, if applicable within the agricultural context, provide another avenue for promoting sustainability by allowing farmers to trade permits for activities that affect the environment. By integrating these policy mechanisms into agricultural governance frameworks, policymakers can foster an environment conducive to the widespread adoption of agroecological practices, thereby advancing the goals of sustainability and environmental conservation.

## 6 Conclusion

The nexus between food self-provisioning and agroecological transitions is not just a trend but also a testament to the resilience and foresight of farmers. As this study highlights, when policies align with the inherent motivations of farmers, the journey towards sustainable agriculture becomes a shared vision rather than a dictated mandate. Investing in this understanding, both in terms of research and policy-making, holds the promise of a future where agriculture thrives in harmony with nature, ensuring food security for all.

The study elucidated the profound connection between food self-provisioning and the migration towards agroecological practices. It became evident that when farmers are anchored in a system where their primary sustenance hinges on their own produce, there emerges a natural inclination towards ensuring the health and longevity of their soil and surrounding ecosystem. Such a paradigm not only fosters sustainable practices but also nurtures an environment where agriculture coexists harmoniously with nature. As the world grapples with the challenges of climate change, dwindling natural resources, and increasing food demands, the results of this study spotlight the necessity of understanding and supporting farmer-driven solutions. By recognizing the intrinsic motivations of farmers, such as the need for self-provisioning, policymakers can craft strategies that resonate on a grass-root level, ensuring higher success rates and tangible positive outcomes.

The significance of this study stretches beyond mere data points and enters the territory of foundational shifts in how we perceive agricultural sustainability. In an era where rapid industrialization often overshadows traditional wisdom, this research reintroduces the timeless bond between a farmer and their land, illuminating the inherent knowledge and instincts that drive sustainable practices. By elucidating the symbiotic relationship between self-provisioning and agroecological transitions, the study beckons policymakers, researchers, and global stakeholders to recenter their strategies around the farmer's intrinsic motivations. Such a perspective, anchored in real-world practices and reinforced by empirical data, offers a beacon for sustainable agricultural frameworks that are not only ecologically viable but also culturally resonant and economically beneficial.

While the current study provides valuable insights into the gender dynamics of food self-provisioning in rural households, it is essential to acknowledge its limitations and scope. One limitation is the focus on a specific geographical area or community, which may restrict the generalizability of the findings to other regions or contexts. Additionally, the study primarily relies on self-reported data, which could introduce biases or inaccuracies based on respondents’ perceptions or recall abilities. Furthermore, the scope of the study is confined to examining gender disparities in crop retention for household consumption, overlooking other factors that may influence food security and dietary diversity, such as income levels, access to markets, or cultural preferences. Future research could address these limitations by employing more diverse sampling methods, incorporating objective measures of food consumption, and exploring broader contextual factors that shape household food provisioning practices.

## Data availability statement

The data that support the findings of this study are openly available in figshare at https://figshare.com/s/58deb8200b1c75cc6543 (https://doi.org/10.6084/m9.figshare.21076312).

## CRediT authorship contribution statement

**ChinasaOnyenekwe:** Investigation, Conceptualization. **Chukwuma Ume:** Methodology, Formal analysis, Data curation. **Ebele Amaechina:** Supervision. **Nice Chukwuma Ume:** Methodology, Formal analysis, Data curation. **Ogochukwu Onah:** Visualization, Validation, Project administration. **Angela Obetta:** Writing – original draft. **Ejiofor Omeje:** Software, Resources.

## Declaration of competing interest

The authors declare the following financial interests/personal relationships which may be considered as potential competing interests. Chukwuma Ume reports financial support was provided by Foundation fiat panis. If there are other authors, they declare that they have no known competing financial interests or personal relationships that could have appeared to influence the work reported in this paper.
