# Supplementary material for: Food self-provisioning: Implications for sustainable agroecological transition in rural Nigeria
Source: Heliyon. 2024 Jun 11;10(12):e32098. doi: 10.1016/j.heliyon.2024.e32098 (PMC11226768; doi:10.1016/j.heliyon.2024.e32098)
Supplement: Multimedia component 1 [file mmc1.pdf]

Household survey questionnaire

JUSTUS-LIEBIG-UNIVERSITÄT GIESSEN

Dear Sir/Ma

My name is Chukwuma Otum Ume, a PhD student from the University of Giessen, Germany. I am carrying out a research project on ‘agroecology and sustainable food systems in Nigeria: agency and food security implications. This questionnaire is meant to help me collect data and information that are necessary for the successful completion of this research work, and you have been selected as one of the respondents. The survey will not take more than 30 minutes. All information provided by you is purely for academic purpose and will be treated confidentially, and strictly anonymously. Thank you.

Direction: Tick (√) or complete the appropriate spaces below for the corresponding question

	**Control question**
1.	Are you the head of the household? Yes [1] No (Continue only if yes)
2.	Are you a member of any agroecology group Yes [1] No [2]
3.	Agricultural Zone [ ]
4.	Name of community [ ]
5.	Enumerator identity number [ ]

PART A: Agroecology indicators		
	**Yes**	**NO**
Crop rotation		
Mixed cropping		
Strip cropping		
Shifting cultivation		
Fallowing		
Cover cropping		
Organic farming		
Mulching		
Reduced or zero tillage		
Agroforestry		
Water harvesting		
Terraces		
Others…		

Relationship with peers		
**Indicators**	**Survey questions**	**Numerical conversion**
credit facilities	Did you borrow any money before?<br><br>From whom?	yes = 1; otherwise = 0<br><br>Government = 1, Cooperatives = 2,<br>Co farmers = 3, Relatives = 4, Banks = 5
Labour sharing	What is the source of labour employed in your farm?	Outsourced labour = 1<br>Family labour = 2<br>Labour sharing (cooperatives or women groups) = 3
educational attainment in years	Please how many years education do you have?	In years
number of extension contact in the last planting season	Please in the last planting season, how many times have any extension officer visited you?	In years
belonging to any peer-to-peer peasant groups	Please do you belong to any peer-to-peer group?	Yes = 1. Otherwise = 0
Relationship with market		
Access to markets	How long does it take you to get to the market?<br><br>Do you sell all your harvest yourself?	In minutes (inverse)<br><br>Self = 1; otherwise = 0

	To what extent do you influence the price?	little extent = 0; not much = 1; large extent = 2;
Access to transportation	Which of these means of transportation do you use?	car = 5, tricycle = 4, bike = 3, public transport = 2, no access = 0)
Trust	How do you establish trust with your customers?	No trust = 0<br>Advertising = 1<br>Personality = 2

PART B: Food Insecurity Experience Scale

Now I would like to ask you some questions about food. During the last 12 MONTHS, was there a time when…:

(Q1)	…you were worried you would not have enough food to eat because of a lack of money or other resources?	YES ☐<br>NO ☐
(Q2)	… you were unable to eat healthy and nutritious food because of a lack of money or other resources?	YES ☐<br>NO ☐
(Q3)	… you ate only a few kinds of foods because of a lack of money or other resources?	YES ☐<br>NO ☐
(Q4)	… you had to skip a meal because there was not enough money or other resources to get food?	YES ☐<br>NO ☐
(Q5)	… you ate less than you thought you should because of a lack of money or other resources?	YES ☐<br>NO ☐
(Q6)	… your household ran out of food because of a lack of money or other resources?	YES ☐<br>NO ☐
(Q7)	… you were hungry but did not eat because there was not enough money or other resources for food?	YES ☐<br>NO ☐
(Q8)	… you went without eating for a whole day because of a lack of money or other resources?	YES ☐<br>NO ☐

PART C: Now I would like to ask you about the types of foods that you or anyone else in your

**household ate yesterday during the day and at night.**<br>Read the list of foods. Place a *one* in the box if anyone in the household ate the food in question; place a *zero* in the box if no one in the household ate the food.		
QUESTIONS AND FILTERS		CODING CATEGORIES
A	Any Garri, bread, rice noodles, biscuits, or any other foods made from millet, sorghum, maize, rice, wheat, or agidi, akamu, asusu, abacha?	(Yes, No: 1,0) .... |__|
B	Any potatoes, yams, manioc, cassava or any other foods made from roots or tubers?	(Yes, No: 1,0) .... |__|
C	Any vegetables?	(Yes, No: 1,0) .... |__|
D	Any fruits?	(Yes, No: 1,0) .... |__|
E	Any beef, pork, lamb, goat, rabbit wild game, chicken, duck, or other birds, liver, kidney, heart, or other organ meats?	(Yes, No: 1,0) .... |__|
F	Any eggs?	(Yes, No: 1,0) .... |__|
G	Any fresh or dried fish or shellfish?	(Yes, No: 1,0) .... |__|
H	Any foods made from beans, peas, lentils, or nuts?	(Yes, No: 1,0) .... |__|
I	Any cheese, yogurt, milk or other milk products?	(Yes, No: 1,0) .... |__|
J	Any foods made with oil, fat, or butter?	(Yes, No: 1,0) .... |__|
K	Any sugar or honey?	(Yes, No: 1,0) .... |__|

**PART D: Agency Variables**<br>Now I would like to ask you questions about your production and sales status		
Land size and property regime	Please could you give me the size of your land (ha) total?<br><br>How much of this land (ha) is cultivated?<br><br>Please can I see your land contract? *(in the absence of land contract, farmer can describe the nature of land ownership)*	Ownership = **5**; in family contract or cooperatives = **4**, Under external contract = **3**; farming only on rented land = **2**
Production diversity	Please list the crops you produce in your fam	

L	Any other foods, such as condiments, coffee, tea?	(Yes, No: 1,0) .... |__|

Level of commercialization	In last planting season, what quantity of the above listed crop did you harvest?<br><br>How much of the harvest did you sell to market?	**A bag = 50kg**

PART E: SOCIOECONOMIC DATA

**Please in this part, I would like to ask you some personal questions, feel free to quit anytime you want.**	
	**Demographic data**
1	Name of community [ ]
2	Gender Male [1] Female [0]
3	In which year where you born? [ ]
4	How many adults are living in this household? (18 years and above) [ ]
5	How many children are living in this household? (Less than 18 years) [ ]
6	What is your marital status? Married [1] Single [2] Divorced [3] widowed [4]
7	If married, how many spouses? [ ]
8	Which of the category below best describe your level of education?<br>Tertiary education [1] Secondary education [2]<br>Primary education [3] No formal education [4]
	**Farm data**
9	How long have you been farming? [Years]
10	Can you estimate the total size of your farm? [Ha]
11	How much of it is: Owned by you [Ha] Leased [Ha]<br>Rented [Ha] Communally owned [Ha]

12	Can you estimate your annual gross farm income?	(₦) Naira
13	What type of crop do you plant? Arable crops 1 Vegetable crops 2<br>Permanent crops 3 Mixed crops 4	
14	What type of cropping system are you practicing? Sequential cropping 3<br>Intercroping 1 Relay cropping 4<br>Monocropping 2 Strip cropping 5	

Thank you very much for your time
